# Infective Endocarditis due to Non-HACEK Gram-Negative Bacilli: Clinical Characteristics and Risk Factors from a Prospective Multicenter Brazilian Cohort

**DOI:** 10.3390/tropicalmed8050283

**Published:** 2023-05-17

**Authors:** Leonardo Paiva de Sousa, Cláudio Querido Fortes, Paulo Vieira Damasco, Giovanna Ianini Ferraiuoli Barbosa, Wilma Felix Golebiovski, Clara Weksler, Rafael Quaresma Garrido, Rinaldo Focaccia Siciliano, Cristiane da Cruz Lamas

**Affiliations:** 1 Instituto Nacional de Cardiologia, Rio de Janeiro 22240-006, Brazil; 2 Instituto Nacional de Infectologia Evandro Chagas, Fundação Oswaldo Cruz, Rio de Janeiro 21040-900, Brazil; 3 Serviço de Doenças Infecciosas e Parasitárias, Universidade Federal do Rio de Janeiro (UFRJ), Rio de Janeiro 21941-901, Brazil; 4 Serviço de Doenças Infecciosas e Parasitárias, Universidade do Estado do Rio de Janeiro (UERJ), Rio de Janeiro 20551-030, Brazil; 5 Departamento de Doenças Infecciosas, Universidade Federal do Estado do Rio de Janeiro (Unirio), Rio de Janeiro 20270-004, Brazil; 6 Instituto do Coração (InCor), Universidade de São Paulo, São Paulo 05403-900, Brazil

**Keywords:** infective endocarditis, gram-negative bacilli, non-HACEK, healthcare-associated infection, central venous catheter, prosthesis, intracardiac devices, hemodialysis

## Abstract

**Background:** Non-HACEK Gram-negative bacilli (NGNB) infective endocarditis (IE) has a growing frequency. We aimed to describe cases of NGNB IE and find associated risk factors. **Methods:** We conducted a prospective observational study of consecutive patients with definitive IE according to the modified Duke criteria in four institutions in Brazil. **Results:** Of 1154 adult patients enrolled, 38 (3.29%) had IE due to NGNB. Median age was 57 years, males predominated, accounting for 25/38 (65.8%). Most common etiologies were *Pseudomonas aeruginosa* and *Klebsiella* spp. (8 episodes, 21% each). Worsening heart failure occurred in 18/38 (47.4%). Higher prevalence of embolic events was found (55,3%), mostly to the central nervous system 7/38 (18.4%). Vegetations were most commonly on aortic valves 17/38 (44.7%). Recent healthcare exposure was found in 52.6% and a central venous catheter (CVC) in 13/38 (34.2%). Overall mortality was 19/38 (50%). Indwelling CVC (OR 5.93; 95% CI, 1.29 to 27.3; *p* = 0.017), hemodialysis (OR 16.2; 95% CI, 1.78 to 147; *p* = 0.008) and chronic kidney disease (OR 4.8; 95% IC, 1.2 to 19.1, *p* = 0.049) were identified as risk factors for mortality. **Conclusions:** The rate of IE due to NGNB was similar to that in previous studies. *Enterobacterales* and *P. aeruginosa* were the most common etiologies. NGNB IE was associated with central venous catheters, prosthetic valves, intracardiac devices and hemodialysis and had a high mortality rate.

## 1. Introduction

Infective endocarditis (IE) is a serious infection with increasing incidence in recent decades and high rates of morbidity and mortality. In recent years, infections caused by Gram-negative bacteria are on the rise, with a greater dispersion of these bacteria in healthcare-related settings, with high mortality rates and high costs to healthcare institutions [1,2,3].

The reported incidence of Gram-negative IE ranges from 1.3 to 10%. Non-HACEK Gram-negative bacilli (NGNB) (species other than *Haemophilus* species, *Aggregatibacter* species, *Cardiobacterium hominis*, *Eikenella corrodens* and *Kingella* species) are uncommon causes of IE [1,3,4,5].

Due to its infrequent occurrence, estimates of NGNB IE prevalence are variable and depend on the study period and geographic location. In the 1990s, NGNB IE was described in association with intravenous drug use [5,6,7].

More recently, studies have shed new light on the contemporary epidemiology of NGNB endocarditis and they have shown it represents approximately 2% to 6% of all cases of IE. The incidence of Gram-negative infections in general is growing. Main risk factors for IE due to NGNB include liver cirrhosis, heart valve prosthesis and urinary-source bacteremia. Hospitalization and medical procedures, such as implantation of endovascular devices and urinary tract procedures, have been strongly associated with NGNB IE [1,8,9,10,11,12,13]. However, a very recent publication on Gram-negative endocarditis across 13 hospitals in Pennsylvania, USA [14], identified 123 cases through electronic records between April 2010 and December 2021, and found a high proportion of intravenous drug users (52%).

Data on NGNB IE are limited to case reports and case series; so far, no study on the issue has been reported in Brazil. The objective of this study was to describe the clinical features of NGNB IE in a multicenter cohort and risk factors associated with IE mortality.

## 2. Materials and Methods

### 2.1. Patients and Study Design

This prospective observational study included consecutive adult patients with definite IE according to the modified Duke criteria identified in 4 centers in Brazil. Cases of NGNB from these cohorts between January 2006 and December 2019 were retrospectively reviewed. Centers participating in this study were Instituto Nacional de Cardiologia, Hospital Universitário Clementino Fraga Filho and Hospital Universitário Pedro Ernesto, all located in Rio de Janeiro city, and Instituto do Coração, located in São Paulo city. The study was approved by each local Ethics Committee.

### 2.2. Data Collection

Data on patient demographics, risk factors, comorbidities, clinical manifestations, echocardiography and microbiological data, treatment and complications were collected retrospectively from each institutional database.

The diagnosis of IE was established according to the modified Duke criteria [2]. All patients 18 years or older with IE due to NGNB from sites that met the criteria were included.

Regarding a possible focus, information was recorded about the presence of a central intravascular access device in the 30 days preceding the diagnosis of IE, of cardiac implantable electronic devices (CIEDs) in the 60 days prior to diagnosis and valve replacement surgery in the year before diagnosis (early prosthetic valve endocarditis).

IE was classified as native valve endocarditis (NVE), prosthetic valve endocarditis (PVE), or CIED-related.

Clinical cases were categorized as community acquired (CoA), nosocomially acquired (NoA) and non-nosocomial healthcare-associated (HCA). NoA endocarditis was defined as IE developing in a patient hospitalized for more than 48 h before onset of signs and symptoms consistent with IE. HCA was defined as IE diagnosed based on signs and symptoms appearing within 48 h before hospital admission in a patient with prior healthcare contact. Acute and subacute IE were defined as an infection with onset of signs and symptoms for less or more than 4 weeks respectively and persistent bacteremia was defined as the persistence of positive blood cultures after 3 days of appropriate antibiotic therapy [15]. Worsening heart failure was defined as clinical and echocardiographic deterioration of heart function.

The microbiological data with identification and resistance pattern were performed using automated methods such as BacT/Alert (bioMérieux; Marcy-L’Étiole, France) and Vitek 2 (bioMérieux). ESBL-producing (extended-spectrum beta-lactamase) bacteria were defined as microorganisms non-susceptible to third and fourth cephalosporin and MDR (multidrug resistant) bacteria as microorganisms non-susceptible to at least one agent in three or more different antimicrobial categories [16,17].

### 2.3. Statistical Analysis

Continuous variables are presented as medians with 25th and 75th percentiles. Categorical variables are presented as frequencies and percentages of the specified group. Univariate comparisons were made with the χ^2^-test. Proportion test (Fisher’s exact test) was applied to analyze risk factors associated with mortality among patients with NGNB endocarditis.

Statistical analyses were performed using commercially available software (R, version 3.5.3, and Jamovi, version 1.1.6.0, Sydney, Australia).

## 3. Results

Of the 1154 patients included, 38 (3.29%) had definitive NGNB endocarditis according to the modified Duke criteria during the study period. There was a male predominance (65.8%, 25/38) and median age was 57 (IQR 43–69) years. The baseline characteristics with comorbidities and predisposing factors are summarized in Table 1. Heart failure (HF) was the most common comorbidity (50%, 19/38), followed by chronic kidney disease (CKD). Prevalence of pre-existing valvular disease (63.2%, 24/38) and an indwelling central venous catheter (34.2%, 13/38) was high.

Native valve IE was present in 16/38 (42.1%) patients. Nineteen patients (50%, 19/38) had prosthetic valve and eight (21.1%, 8/38) had CIED-related IE due to NGNB. The patients were more likely to be affected by NoA IE (52.6%, 20/38) and HCA IE (26.3%, 10/38).

As shown in Table 2, the most common IE lesions were vegetations. Most patients had endocarditis localized on the aortic valve (44.7%, 17/38), followed by the mitral valve (42.1%, 16/38) and CIED-related IE (21.1%, 8/38). Native valves only were affected in twelve (31.6%), prosthesis only in eighteen (47.3%), intracardiac devices only in four (10.5%), native valves and prosthesis in two (5.3%) and native valves and intracardiac devices in two (5.3%).

All patients had definite IE according to the Duke criteria. Major microbiological Duke criteria were present in 32/38 (84.2%) patients, of which 10/32 (31.3%) had positive blood cultures for Gram negatives more than 12 h apart, and 22/32 (68.8%) had all of three or a majority of four or more separate blood cultures positive for Gram negatives (with the first and the last samples drawn ≥ 1 h apart). In the remaining six patients, diagnosis was based on a major echocardiographic criterion and at least three minor criteria.

Patients with IE due to NGNB had a high prevalence of previous heart valve replacement surgery (57.9%, 22/38) and an indwelling central intravascular access device (34.2%, 13/38) at the time or within 30 days of IE onset.

The most common symptom was fever (57.9%, 22/38). Embolic events were observed in twenty-one patients (55.3%, 21/38). The main sites for embolic events were the brain (*n* = 6) and spleen (*n* = 5). Erythrocyte sedimentation rate (ESR) and C-reactive protein (CRP) were elevated in 8/38 (21.1%) and 29/38 patients (76.3%), respectively.

Central venous catheters and recently implanted valve prosthesis were the major presumed sources of infection, accounting for 11/38 (28.9%) and 9/38 (23.7%) of cases, respectively, as shown in Table 3.

Microbiological spectrum and drug resistance of IE due to NGNB in the study population are detailed in Table 4. In our study the most common etiologies were *Klebsiella* spp. (21%, 8/38), *Pseudomonas aeruginosa* (21%, 8/38) and *Serratia marcescens* (16%, 6/38).

Table 5 describes the antibiotic regimens received by 35 patients with NGNB endocarditis where this information was available. Most patients (42.8%, 15/35) received an antibiotic regimen containing a non-carbapenem beta-lactam, mostly cefepime and piperacillin-tazobactam, variably combined with an aminoglycoside or a fluoroquinolone. A substantial number of cases (48.6%, 17/35) were treated with a carbapenem with or without other antibiotics (including polymyxin). Combined antimicrobial regimens with amikacin and gentamicin were used in 4/35 (11.4%) and 6/35 (17.1)%, respectively.

Among 38 patients with NGNB IE complications, 18 cases (47.4%) presented worsening heart failure.

Embolic events were the most common complication. IE complications are described in Table 6.

Cardiac surgical procedure was performed in eight (21%) patients. Of the 38 patients with IE due to NGNB, there were 19 deaths (50%). When comparing non-fermenting Gram negatives with enterobacterales, mortality was 11/15 (73%) vs. 8/23 (34.7%), with a *p* value = 0.045 (Fisher’s exact test). When we compared patients who had valve surgery for NGNB IE with those who only had antibiotic treatment, we found 8/19 (42%) surgically treated died, vs. 11/19 (57.9%) of those treated conservatively (p NS).

As shown in Table 7, indwelling CVC (*p* = 0.017) and chronic kidney disease with haemodialysis (*p* = 0.003) were identified as risk factors associated with mortality.

## 4. Discussion

This is the first Brazilian series describing the characteristics and risk factors associated with mortality of patients affected by IE due to NGNB. Due to its infrequent occurrence, estimates of NGNB IE prevalence are variable and depend on the study period. Such population selection bias also restricted earlier studies to large metropolitan cities. The earliest systematic review of NGNB IE, conducted from 1945 to 1977, concentrated on intravenous drug users [18].

The incidence of IE cases due to NGNB varies in the literature, ranging from 2% to 6%, with recent series showing higher incidences. These growing numbers are probably related to the presence of cardiac and vascular devices, invasive medical procedures, recurrent hospitalization, and immunosuppression [1,8,9,10,11,12,13]. Previous studies showed that IE due to NGNB was predominantly on the right side of the heart, especially in cases associated with the use of injectable drugs [6].

Over the decades with the increase of IE caused by NGNB, the pattern of the infection has also changed, with the left side of the heart being the most affected nowadays. Left-sided infective NGNB endocarditis has some features such as the following: the course of the disease has a median onset of symptoms of 15 days and high rates of complications, including congestive heart failure, perivalvular abscesses, and peripheral, splenic and central nervous system embolization [6,11]. Although investigators from the ICE group showed that most cases of IE due to NGNB had a subacute diagnosis [1], our data show that in 84.2% of the cases the diagnosis was made within 30 days of the onset of symptoms.

Fever (81.6%) was the most common clinical finding and is described as one of the first signs/symptoms in IE due to NGNB [10]. Of the other minor Duke criteria [2], we found pre-existing valve disease in 63.2% of cases. Among the findings that can increase the sensitivity of the diagnosis of IE [19], the St. Thomas’ suggested minor criteria, our study showed elevated CRP in 76.3%, elevated ESR in 21.1%, hematuria in 13.2% and indwelling CVC in 34.2% of cases of NGNB endocarditis.

In our study the mitral and aortic valves were more often affected, and we had no intravenous drug users at all in our sample. All right-sided NGNB IE in our series was related to intracardiac devices. A very recent publication from the United States shows a high prevalence of intravenous drug users in a contemporary series of 123 patients with Gram-negative IE [14].

There are few case series reporting IE due to NGNB and Table 8 summarizes the main findings from them.

The incidence of NGNB endocarditis in our study was 3.29% and was similar to the incidence in a large international series previously published. The main clinical findings were HF in 19 (50%), CKD in 17 (44.7%), ACD in 10 (26.3%) and hemodialysis in 10 (26.3%) patients. Male sex was the most affected (65.8%) with a median age of 57 years (IIQ 43–69). Age and gender have been reported as important risk factors in IE due to NGNB. Historically, elderly males are the most affected population [1,10,11,12].

Non-fermenting bacteria is the main group of non-HACEK Gram-negative bacilli, NGNB, reported in the literature as causing IE. This group includes species as *Pseudomonas aeruginosa*, *Acinetobacter* sp., *Burkholderia cepacia* and *Stenotrophomonas malthophilia* [6,7,20]. In a recent epidemiological study in the United States, this bacterial group corresponded to 70% of IE due to NGNB and *Pseudomonas aeruginosa* (68%) was the main etiological agent [11].

Among the group of fermenting GNBN that causes IE, the *Enterobacteriaceae* family is the most relevant, and *Klebsiella* spp. and *Enterobacter cloacae* were the most common agents. The International Collaboration on Endocarditis (ICE) study [1], and more recently an Argentine case series [9] and an Italian cohort [10], showed that *Escherichia coli* was the main microbiological agent of IE due to NGNB; in their studies, this is probably related to high rates of urinary bacteremia and high rates of use of urinary catheters.

On the other hand, a recent North American publication [14] in which intravenous drug users comprised over half the cases showed *S. marcescens* as the most frequently isolated Gram negative (43%).

In our series, enteric Gram-negative bacilli were the most frequent etiology as a group. This relates to their frequency as etiologies of nosocomial infection. *Klebsiella* sp. accounted for 21% and *Serratia marcescens* for 6%. *P. aeruginosa*, a non-fermenter, accounted for 21%. These etiological agents are the main NGNB identified in central venous catheter-related bacteremia in Brazil, which is consistent with the main risk factors of IE due to NGNB acquisition found in this study: the presence of a central venous catheter (34.2%) and patients undergoing hemodialysis (26.3%). This probably indicates a problem with infection control. The NGNB, except for *Salmonella* spp. and *Pseudomonas aeruginosa*, have limited capacity for biofilm formation and low capacity for adhesion to the endocardium [21]. However, the presence of prosthesis and of intracardiac devices facilitate adherence and the formation of vegetations.

The structures most affected in the 38 cases of IE due to NGNB were prosthetic valves (50%). The incidence of NGNB endocarditis in CIED and prosthetic valves has increased over the years due to previous healthcare contact and the permanence of these patients in hospital institutions [1,22]. When we performed a subgroup analysis comparing cases of NGNB IE with other etiologies in a cohort of adults with definite IE, in one of the participating centers (INC), we found that early prosthetic valve endocarditis and the presence of intracardiac devices were significantly associated with the former, as was hospital acquisition. Clinical features were similar except for a significantly higher proportion of paravalvular abscess and persistent bacteremia (Lamas C, unpublished data).

Current guidelines recommend the use of antibiotics for 6 weeks [22,23,24]. Mechanisms of antimicrobial resistance in NGNB have increased dramatically across the planet [25,26], making this a public health issue [27]. A single-center retrospective study involving 60 patients with Gram-negative infective endocarditis, found by ICD coding in the years 2011 to 2019 in Ohio, USA, attempted to answer the query on whether combination therapy was superior to monotherapy [28]. The frequency of intravenous substance abuse was high, 21/60 (35%). There was no difference in 60-day mortality, which was 7/34 (21%) in patients who had monotherapy vs. 5/26 (19%) of those on combination therapy [28]. This publication had small numbers of patients and a retrospective design, which means severity of illness at presentation may have affected treatment allocation, as there was more intravenous drug use, CNS involvement and metastatic sites at baseline in the combination therapy group.

**Table 8:** Main findings from the IE due to Non-HACEK Gram-negative bacilli.

Author, Year	Study Period	Study Site	*N* (Patients with IE Due to NGNB)	NoA IE/HCA IE	Age in Years Median (IQR)	Male	Most Common Etiological agent	Type of IE	Surgical Treatment	Mortality
Morpeth et al., 2007 [1]	2000–2005	International multicenter	49/2761 (1.8%)	18/46 (39%)/8/46 (17%)	63 (50–71)	29/49 (59%)	*E. coli* 14/49 (28%) and *P. aeruginosa* 11/49 (22%)	NVE 20/49 (41%), PVE 29/49 (59%), CIED N/A	25/49 (51%)	12/49 (24%)
Mercan et al., 2019 [8]	2007–2016	Multicenter, Turkey	26	16/26 (61%)	53 (28–84)	11/26 (42%)	*P. aeruginosa* 7/26 (27%) and *E. coli* 7/26 (27%)	NVE 21/26 (81%), PVE 5/26 (19%), CIED 1/26 (4%)	10/26 (38%)	6/26 (23%)
Burgos et al., 2018 [9]	1998–2016	Single center, Argentina	24/355 (6.7%)	N/A	72 (N/A)	17/24 (71%)	*E. coli* 6/24 (25%) and *P. aeruginosa* 5/24 (21%)	NVE 6/24 (25%), PVE 11/24 (45.8%), CIED 7/24 (29%)	9/24 (37%)	5/24 (21%)
Falcone et al., 2018 [10]	2004–2011	Multicenter, Italy	58/1722 (3.3%)	24/58 (41%)/2/58 (3%)	69.5 (57.75–77)	39/58 (67%)	*E. coli* 18/58 (31%) and *Pseudomonas* sp. 11/58 (19%)	NVE 34/58 (59%), PVE 16/58 (28%), CIED 8/58 (13%)	25/58 (43%)	8/58 (14%)
Veve et al., 2020 [11]	2011–2019	Single center, USA	43	N/A	40 (31–50)	22/43 (51%)	*P. aeruginosa* 30/43 (68%) and *S. marcescens* 9/43 (20%)	NVE 30/43 (70%), PVE 13/43 (30%), CIED 2/43 (1%)	10/43 (23%)	20/43 (47%)
Trifunovic et al., 2018 [12]	2008–2015	Single center, Serbia	9/246 (3.7%)	N/A	N/A	N/A	*P. aeruginosa* 4/9 (44%) and *K. pneumoniae*,	N/A	N/A	N/A
Loubet et al., 2015 [13]	2009–2014	Single center, France	12/300 (4%)	2/12 (17%)/2/12 (17%)	51 (44–74)	8/12 (66%)	*E. coli* 4/12 (33%) and *P. aeruginosa* 3/12 (25%)	PVE 8/12 (67%), NVE e CIED 0/12 (0%)	7/12 (58%)	1/12 (8%)
Shah et al., 2023 [14]	2010–2021	Multicenter in a single state (Pennsylvania), USA	123	64/123 (52%) *	49 (32–66)	77/123 (63%)	*Serratia* spp. (43%; 53/123), *P. aeruginosa* (21%; 26/123) and *Klebsiella* spp. (14%; 17/123) (	PVE 21/123 (17%), CIED 13/123 (10%)	84/123 (68%)	25 (20%)
Lorenz et al., 2021 [28]	2011–2019	Single center, Ohio, USA	60/1036 (5.8%)	N/A	49.5 (35.5–61.5)	40 (67%)	*P. aeruginosa* 22/60 (37%), *Serratia* 12/60 (20%), *E.coli* 10/60 (17%)	PVE 15 (25%), CIED 12(20%)	11/60 (18.3%)	12/60 (20%)

*IE* infective endocarditis; *NGNB* non-HACEK Gram-negative bacilli; *NoA IE* nosocomially infective endocarditis; *HCA IE* non-nosocomial healthcare-associated; *IQR* interquartile range; *NVE* native valve endocarditis; *PVE* prosthetic valve endocarditis; *CIED* cardiac implantable electronic device; *N/A* not available. * Persons who inject drugs.

Historically, beta-lactams with or without aminoglycoside regimes, whether or not associated with fluoroquinolone, are the drugs of choice for the treatment of endocarditis caused by NGNB. These recommendations are based on expert opinions and are debatable; a recent series showed no difference for patients who received combination therapy (n = 53) compared to those treated with monotherapy regarding median lengths of stay (23 vs. 19.5 days; *p* = 0.412), microbiologic failure rates (11.3% vs. 7.1%; *p* = 0.528), clinical failure rates (18.9% vs. 22.9%; *p* = 0.592) and 90-day mortality rates (13.2% vs. 25.7%; *p* = 0.088). However, this same study [28] showed in the subgroup of 89 patients with GNIE due to *Enterobacterales* that rates of clinical failure were numerically, but not statistically, lower among those who received combination (n = 31) vs. monotherapy (n = 58; 10% vs. 26%, respectively; *p* = 0.09). For these patients, 90-day mortality rates were also numerically lower for patients who received combination therapy vs. monotherapy (15% vs. 41%; *p* = 0.037).

Due to the varied profile of possible etiologic agents and drug resistance, indications for the treatment of IE due to MDR NGNB are still debated, because of the high risk of clinical failure. In these cases, antibiotic therapy should be individualized, combination therapy should be provided if possible, and a consultation for the indication of prompt surgical removal of infected valves should be performed. Despite all, MDR in NGNB in our study was not related to mortality and was not related to carbapenem-including regimes.

Surgical intervention in previous cohorts varies from 23 to 58% [1,9,10]. Although previous studies have shown benefits in combined clinical and surgical treatment, some authors reported no significant difference in outcome between clinical treatment alone versus combined medical and surgical intervention [6,29]. In our small sample, we did not find a difference in mortality for our patients regarding surgery. In a recent North American study, in multivariable logistic regression analysis for patients with GNIE due to *Enterobacterales*, only no surgery despite a surgical indication (OR, 31.1; 95% CI, 6.08–159; *p* < 0.001) and age (OR, 1.07 per year; 95% CI, 1.02–1.11; *p* = 0.002) were independently associated with 90-day mortality [14]. This could perhaps be explained by the introduction of new antimicrobial medications against NGNB in the last decades.

Previous data on IE mortality due to NGNB show rates between 8% and 47% [1,9,10,11]. In our study, mortality due to NGNB endocarditis was significantly high (50%, 19/38). Mortality was even higher when non-fermenters were the cause of NGNB, when compared to *Enterobacterales*. Of interest, MDR etiology was not a risk factor associated with mortality. On the other hand, indwelling CVC and chronic hemodialysis were associated with mortality among patients affected by NGNB endocarditis.

Our study has some limitations. First, because of the rarity of infection, we were able to investigate only a small number of patients overall. Moreover, the study was conducted at referral centers (two university hospitals and two cardiac surgery referral institutes), where the complicated cases of IE might be overrepresented. The major strength of our study is that it represents the first Brazilian contemporary study describing IE due to NGNB. This is a multicenter prospective study, which increases the value of the results obtained.

## 5. Conclusions

In conclusion, healthcare contact is an emerging risk factor for NGNB endocarditis. All patients with NGNB IE should be managed in consultation with specialists. Prosthesis and endovascular devices as well as intravenous catheters and chronic renal disease were highly prevalent in NGNB endocarditis in our contemporary multicenter cohort. Mortality was associated with the presence of central catheters and with hemodialysis. The frequency of NGNB IE, antimicrobial and surgical treatment strategies, and the role of antibiotic resistance on outcome remain to be evaluated in future studies.

## Figures and Tables

**Table 1:** Baseline characteristics and predisposing factors of patients with Non-HACEK Gram-negative bacilli endocarditis.

Variable	*n* = 38
Age, median years (IQR)	57 (43–69)
Male sex	25 (65.8%)
Heart Failure	19 (50%)
Arterial Coronary Disease	10 (26.3%)
Rheumatic Heart Disease	6 (15.8%)
Congenital Heart Disease	0 (0%)
Chronic Kidney Disease	17 (44.7%)
Hemodialysis	10 (26.3%)
Smoking	6 (15.8%)
Chronic Obstructive Pulmonary Disease	2 (5.3%)
Diabetes	7 (18.4%)
Autoimmune disease	1 (2.6%)
HIV-1 infection	0 (0%)
Malignancies	1 (2.6%)
Pre-existing valvular disease	24 (63.2%)
Central venous catheter	13 (34.2%)
Native Valve	16 (42.1%)
Prosthetic Valve	19 (50%)
Cardiac Implantable Electronic Device	8 (21.1%)
Immunosuppression	2 (5.3%)
Previous infective endocarditis	3 (7.9%)
Intravenous drug user	0 (0%)

*IQR* interquartile range.

**Table 2:** Echocardiographic findings of patients with Non-HACEK Gram-negative bacilli IE.

Characteristics	No. of Patients (%)
Aortic valve vegetation	17 (44.7)
Mitral valve vegetation	16 (42.1)
Tricuspid valve vegetation	4 (10.5)
CIED-related IE	8 (21.1)
New valvular regurgitation	5 (13.2)
Perivalvar abscess	1 (2.6)
Dehiscence of prosthetic valve	1 (2.6)
Perivalvular pseudoaneurysm	2 (5.2)
Perforation	4 (10.5%)
Median (range) size of vegetation (mm)	11 (5–21)

*CIED* cardiac implantable electronic device; *IE* infective endocarditis.

**Table 3:** Presumed source of infection of patients with Non-HACEK Gram-negative bacilli IE.

Presumed Source of Infection	No. of Patients (%)
Central venous catheter	11 (28.9)
Prosthetic valve *	9 (23.7)
CIED	5 (13.2)
Genitourinary tract	2 (5.2)
Skin	1 (2.6)
Gastrointestinal tract	2 (5.3)
Respiratory tract	2 (5.3)
Other or unknown	6 (15.8)

*CIED* cardiac implantable electronic device; *IE* infective endocarditis. * 1 prosthetic and CIED co-infection.

**Table 4:** Etiologies of patients with Non-HACEK Gram-negative bacilli IE.

Etiologies	No. of Patients (%)
***Enterobacterales***	
*Klebsiella* spp.	8 (21)
*Serratia marcescens*	6 (16)
*Enterobacter* spp.	4 (10)
*Escherichia coli*	2 (5)
*Salmonella* spp.	2 (5)
*Citrobacter* spp.	1 (3)
**Non-fermenters**	
*Pseudomonas aeruginosa*	8 (21)
*Burkholderia cepacia*	3 (8)
*Acinetobacter* spp.	3 (8)
*Stenotrophomonas maltophilia*	1 (3)
**Total**	38 (100)
Susceptible *Enterobacterales*	17 (45)
Susceptible *Pseudomonas aeruginosa*	8 (21)
Susceptible non-fermenters	5 (13)
All MDR bacteria *	8 (21)
**Total**	38 (100)

* Comprising extended spectrum beta-lactamase producers, ESBL-producing *Enterobacterales* and multidrug resistant, MDR strains (*Escherichia coli*, *Klebsiella* spp., *Serratia marcescens*, *Citrobacter* spp., *Enterobacter* spp., *Salmonella* spp., *Stenotrophomonas maltophilia*, *Burkholderia cepacia*, *Acinetobacter* spp. strains).

**Table 5:** Treatment strategies for Non-HACEK Gram-negative bacilli IE.

Antibiotic Therapy	*n* = 35
Non-carbapenem beta-lactam ± aminoglycoside ± fluoroquinolone	15 (42.8)
Carbapenem ± aminoglycoside ± fluoroquinolone	10 (28.6)
Carbapenem	6 (17.1)
Fluoroquinolone	2 (5.7)
Co-trimoxazole	1 (2.9)
Polymyxin-containing regime (associated with amikacin and meropenem)	1 (2.9)

**Table 6:** Complications among patients with Non-HACEK Gram-negative bacilli IE.

Complications	*n* = 38
Worsening HF	18 (47.4%)
Worsening kidney function	14 (26.8%)
Persistent bacteremia	6 (15.8%)
Valvular dysfunction	3 (7.9%)
Embolic events	21 (55.3%)

*HF* = heart failure.

**Table 7:** Variables associated to mortality in Non-HACEK Gram-negative bacilli IE.

Variables	Mortality	*p*-Value
Prosthetic valve IE	7/19 (36.8%)	0.105
Acute IE	16/19 (84.2%)	1.0
Diabetes	6/19 (31.5%)	0.036
Heart failure	10/19 (52.6%)	1.000
Chronic kidney disease without HD	3/19 (15.7%)	0.676 *
**Chronic kidney disease with HD**	**9/19 (47.3%)**	**0.003**
Acute kidney failure	7/19 (36.8%)	1.000
Nosocomially-acquired IE	9/19 (47.3%)	0.746
Valvular abscess	2/19 (10.5%)	1.000 *
CNS embolization	5/19 (26.3%)	0.209
Splenic embolization	3/19 (15.7%)	1.000 *
Persistent Bacteraemia	4/19 (21%)	0.660 *
Worsening renal function	7/19 (36.8%)	1.000
Worsening heart function	11/19 (57.8%)	0.194
**CVC**	**10/19 (52.6%)**	**0.017**
Previous Cardiac Surgery	9/19 (47.3%)	0.501
CIED	3/19 (15.7%)	0.693 *
Immunosuppression	2/19 (10.5%)	0.486 *
Previous IE	1/19 (5.2%)	1.000 *
Pre-existing valvular disease	11/19 (57.8%)	0.737
Increased CRP	14/15 (93.3%)	0.621
Susceptible *Pseudomonas aeruginosa*	6/19 (31.5%)	0.111
Susceptible *Enterobacteriaceae*	7/19 (36.8%)	0.328
MDR strains **	2/19 (10.5%)	0.232 *

* *p*-value calculated by Fishers’ exact test; otherwise, *p* value was calculated by the chi-square test. ** MDR=multidrug resistant, as defined in the methods: non-susceptible to at least one agent in three or more different antimicrobial categories [16,17]. IE infective endocarditis, HD haemodialysis, CNS=central nervous system; CVC central venous catheter, CIED cardiac implantable electronic device, CRP C-reactive protein, MDR multidrug resistant.

## Data Availability

Data generated or analyzed in this study have not been made available due to confidentiality issues.
